# Choice impulsivity after repeated social stress is associated with increased perineuronal nets in the medial prefrontal cortex

**DOI:** 10.1038/s41598-024-57599-6

**Published:** 2024-03-26

**Authors:** Christopher A. Martinez, Harry Pantazopoulos, Barbara Gisabella, Emily T. Stephens, Jacob Garteiser, Alberto Del Arco

**Affiliations:** 1 HESRM, School of Applied Sciences, University of Mississippi, Oxford, MS USA; 2 Department of Psychiatry and Human Behavior, Medical School, University of Mississippi Medical Center, Jackson, MS USA

**Keywords:** Social stress, Decision-making, Reward-seeking, Impulsivity, Interneurons, Extracellular matrix, Neuroscience, Psychology

## Abstract

Repeated stress can predispose to substance abuse. However, behavioral and neurobiological adaptations that link stress to substance abuse remain unclear. This study investigates whether intermittent social defeat (ISD), a stress protocol that promotes drug-seeking behavior, alters intertemporal decision-making and cortical inhibitory function in the medial prefrontal cortex (mPFC). Male long evans rats were trained in a delay discounting task (DDT) where rats make a choice between a fast (1 s) small reward (1 sugar pellet) and a large reward (3 sugar pellets) that comes with a time delay (10 s or 20 s). A decreased preference for delayed rewards was used as an index of choice impulsivity. Rats were exposed to ISD and tested in the DDT 24 h after each stress episode, and one- and two-weeks after the last stress episode. Immunohistochemistry was performed in rat’s brains to evaluate perineuronal nets (PNNs) and parvalbumin GABA interneurons (PV) labeling as markers of inhibitory function in mPFC. ISD significantly decreased the preference for delayed large rewards in low impulsive, but not high impulsive, animals. ISD also increased the density of PNNs in the mPFC. These results suggest that increased choice impulsivity and cortical inhibition predispose animals to seek out rewards after stress.

## Introduction

It is well established that a history of stress leads to drug seeking and relapse, which ultimately enhances the risk of developing substance use disorders (SUDs)^1–3^. Therefore, identifying the behavioral and neurobiological mechanisms that drive the *transition* from stress to substance abuse is critical to predict, prevent and treat SUDs. Choice impulsivity is one of the most reliable behavioral features that anticipates the transition to substance abuse and is a common symptom in numerous psychiatric disorders^4–7^. It is a value-based decision-making bias that leads individuals to overvalue small reward choices compared to large rewards that come with a time delay^7,8^. By using delay-discounting tasks (DDTs), behavioral studies have demonstrated that the longer the delay to obtain a large reward, the more that individuals (i.e. humans and experimental animals) shift their preference to a smaller, immediate reward. In this context, *delay discounting* serves as an index of choice impulsivity^7,8^.

Previous studies have shown that stress alters value-based decision making and increases risk-taking behaviors^9–11^. However, only a few of these studies have focused on delay discounting behavior and the results are inconclusive^12^. Thus, in humans, acute psychological stress is reported to both increase^13,14^ and decrease^14^ delay discounting, while in animal models, acute restraint stress does not change this behavior^15^. Also, in animal models, early life stress was reported to not change^16,17^ or increase^18^ delay discounting in adult rats. These studies, most likely due to the different stress protocols utilized, do not clarify the relationship between stress and delay discounting behavior, and most importantly, do not inform whether choice impulsivity could be a link between stress and drug abuse.

Intermittent social defeat (ISD) is a stress protocol in rodents with translational validity that increases the escalation of drug intake and facilitates the emergence of addiction-like behaviors^19,20^. In rats, ISD increases self-administration of psychostimulants (i.e. cocaine, amphetamine) and sugar pellets several weeks after the last stress episode^21–23^, suggesting that ISD promotes reward-seeking behavior in the long term. Here we designed a time sensitive approach to investigate longitudinally whether ISD increases choice impulsivity, which ultimately, could predispose individuals to seek out rewards.

Given the prevalence of impulsivity in psychiatric disorders^4,24^, different studies have investigated the neurobiological substrates that contribute to delay discounting behavior in humans and experimental animals^5,7,25^. Imaging studies find that changes in the function of fronto-striatal pathways are correlated to individual differences in choice impulsivity^26,27^. Animal studies support these findings and show that lesions in the prefrontal cortex and ventral striatum (i.e. nucleus accumbens) alter delay discounting behavior^28,29^. In particular, the medial prefrontal cortex (mPFC) seems to play a key role in this behavior. Thus, in the mPFC, it has been shown that the activation of GABA_B_ receptors decreases delay discounting^30^ while the blockade of D1 and D2 dopamine receptors increase delay discounting^31^ (i.e. increases choice impulsivity). Furthermore, electrophysiological and pharmacogenetic studies demonstrate that specific neuronal populations in the mPFC regulate delay discounting behavior^32,33^. In particular, a recent study shows that the inhibition of mPFC neuronal projections to nucleus accumbens increases choice impulsivity in rats, and that this effect depends on individuals’ basal levels of impulsivity^33^.

Here we hypothesize that ISD increases choice impulsivity and that this effect can be associated with markers of inhibitory function in the mPFC. Previous studies suggest that the exposure to stress alters cortical excitatory/inhibitory balance and that this is causally link to stress-induced behavioral deficits (i.e. depression-like behavior, cognitive flexibility, anxiety)^34–36^. In support of this suggestion, it has been shown that chronic stress increases the number of parvalbumin GABA inhibitory interneurons (PV)^37^ in the mPFC, and that increasing/decreasing the activity of PV cells or the expression of perineuronal nets (PNNs) in the mPFC reverses the behavioral deficits produced by stress^35,38–41^. PNNs are extracellular matrix structures that enhance the inhibitory activity of cortical PV cells^41–44^. Based on this evidence, in the current study we first determined whether ISD alters delay discounting behavior, and second, whether ISD changes cortical inhibitory function. As a first step in examining alterations in mPFC circuitry that may contribute to altered neuronal activity following ISD, we examined two markers of inhibitory neurons, PNNs and PV-immunoreactive neurons, in the mPFC. We focused the current study on male rats in order to compare with previous studies using ISD as well as our own recent work^23,45^.

## Results

### Behavior in the DDT

**Figure 1:**
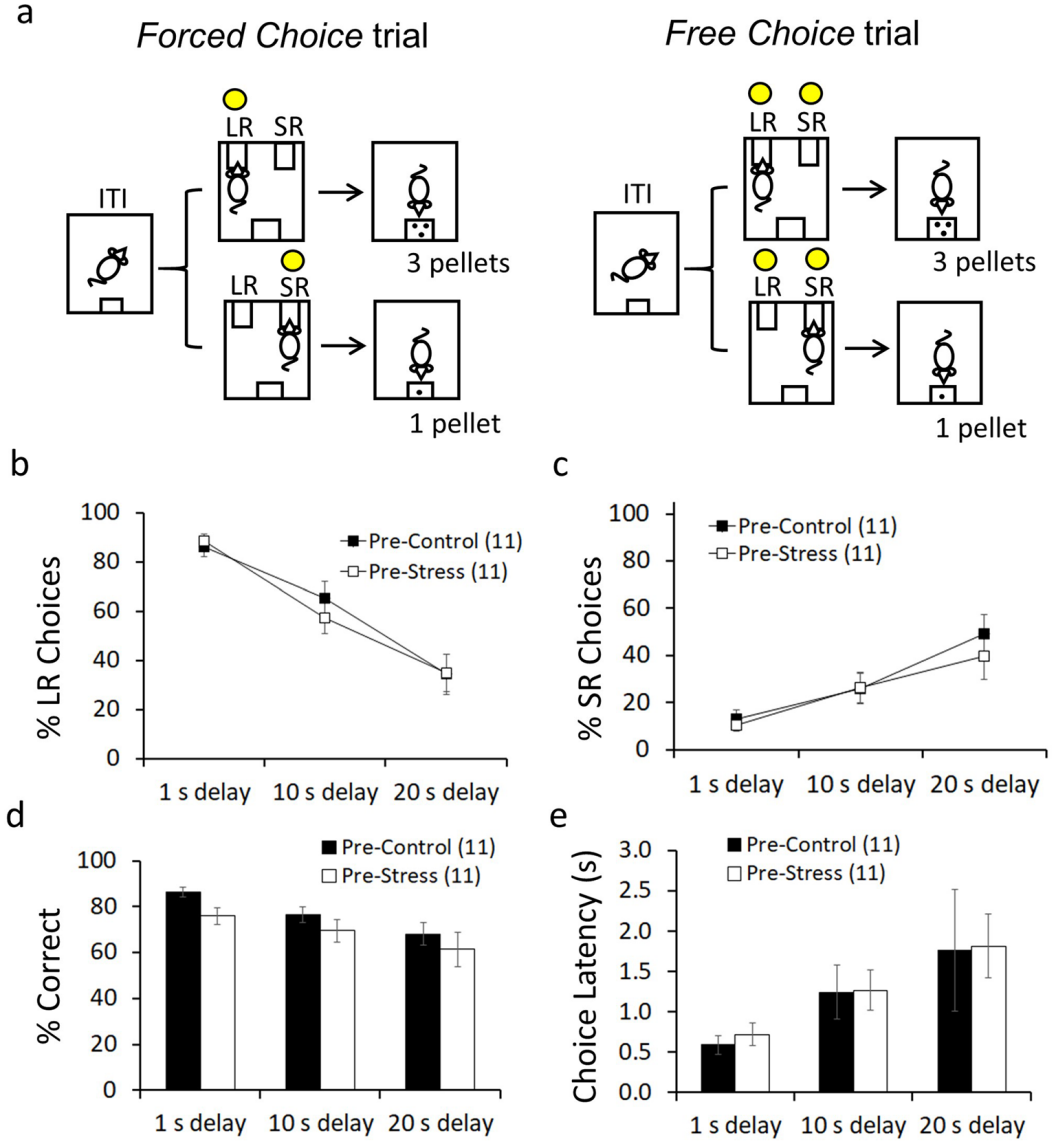
Delay discounting task and behavior. (**a**) Diagram representing *Force Choice* and *Free Choice* trials during the task. In *Forced Choice* trials, the two levers, LR and SR, are extended but only the active lever shows a cue light. In *Free Choice* trials, the two levers are active, and rats need to make a choice. Graphs show LR choices (**b**), SR choices (**c**), accuracy (**d**) and choice latency (**e**) during the delay discounting task at different delays for LR (1 s, 10 s and 20 s) and for all rats before starting the stress protocol. The delay for SR was always 1 s. Data represents the mean ± SEM. The mean values are the average of the last two training sessions for every dependent variable at different delays for LR.

The animals were trained in the DDT (Fig. 1a) until stable performance and then split into two groups, Pre-Control and Pre-Stress. Stable performance was confirmed by two-way ANOVA analysis (see “Methods”) that showed no differences in large reward (LR) choices among the last six training sessions (session: F_(5,100)_ = 0.86, p = 0.506, η_p_^2^ = 0.04) considering group (session x group: F_(5,100)_ = 1.37, p = 0.240, η_p_^2^ = 0.06) and delay (delay × session: F_(10,200)_ = 1.51, p = 0.137, η_p_^2^ = 0.07) as factors. After training, rats in the Pre-Control group were handled while rats in the Pre-Stress group were submitted to ISD (see “Methods”). The number of correct responses (i.e. accuracy) was evaluated during Force Choice trials. The number of LR and small reward (SR) choices (i.e. LR and SR lever presses) and the latency to press the LR lever (i.e. choice latency), were evaluated during the Free Choice trials.

Figure 1 shows that all rats decrease their preference for LR and increase their preference for SR as the delay to LR delivery increases to 10 s and 20 s. Considering the average of the two last training sessions, a two-way ANOVA with repeated measures with group (Pre-Control, Pre-Stress) as between subjects and delay (1 s, 10 s and 20 s) as within subjects was used to analyze performance in the DDT. The results of this analysis show that there is a decrease in the number of LR choices (Fig. 1b) (delay: F_(2,40)_ = 69.69, p < 0.001, η_p_^2^ = 0.77), and an increase in the number of SR choices (Fig. 1c) (delay: F_(2,40)_ = 28.19, p < 0.001, η_p_^2^ = 0.58) with higher delays. They also show that there is a decrease in accuracy (% correct responses in forced choice trials) (Fig. 1d) (delay: F_(2,40)_ = 11.91, p < 0.001, η_p_^2^ = 0.37), that was significant at the highest delay (F_(2,19)_ = 9.12, p = 0.002, η_p_^2^ = 0.49), and an increase in choice latency (Fig. 1e) (delay: F_(2,40)_ = 10.76, p < 0.001, η_p_^2^ = 0.35) with higher delays. No significant differences were found between Pre-Control and Pre-stress groups in LR and SR choices or choice latency.

**Figure 2:**
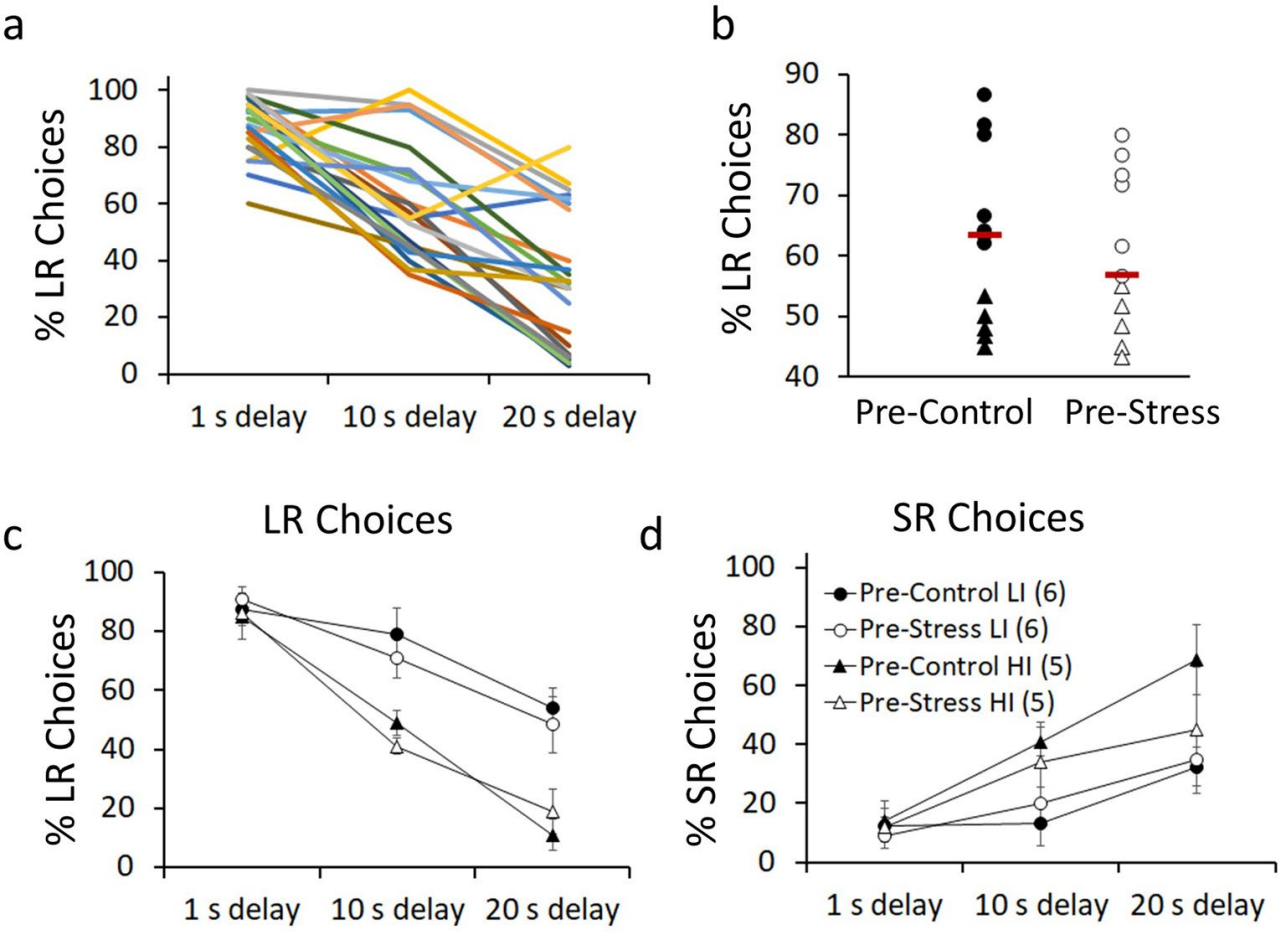
Animals can be divided into low impulsive (LI) and high impulsive (HI) according to their basal performance. (**a**) Individual differences in the preference for LR choices at different delays for LR (1 s, 10 s and 20 s). Every line represents one animal (n = 22). (**b**) A median test was utilized to group animals as LI or HI. Every dot represents one animal as the average of LR choices across delays during the last two training sessions. Red lines indicate median values. (**c)** LR and (**d**) SR choices at different delays for LR comparing LI and HI animals before stress. Data represents the mean ± SEM. The mean values are the average of the last two training sessions.

Based on previous studies^32,46^, the rats were grouped according to their basal levels of impulsivity. A median test was performed on the average number of LR choices across the 3 different delays for LR (1, 10 and 20 s) to group rats into low impulsive (LI) (n = 6 per group) and high impulsive (HI) (n = 5 per group) (Fig. 2a and b). As shown in Fig. 2c, LI rats made more LR choices compared to HI rats with higher delays (delay x impulsivity: F_(2,36)_ = 3.88, p = 0.03, η_p_^2^ = 0.17). Pairwise comparisons showed that LI rats made more LR choices compared to HI rats at 10 s (F_(1,18)_ = 9.98, p = 0.005, η_p_^2^ = 0.35) and 20 s delays (F_(1,18)_ = 11.78, p = 0.003, η_p_^2^ = 0.39), but not at 1 s delay (F_(1,18)_ = 3.47, p = 0.08, η_p_^2^ = 0.16). Figure 2d shows that LI and HI rats were also significantly different in the number of SR choices. Li rats made fewer SR choices than HI rats at higher delays (delay × impulsivity: F_(2,36)_ = 3.58, p = 0.038, η_p_^2^ = 0.16). There were not significant differences between LI and Hi rats in accuracy (delay × impulsivity: F_(2,36)_ = 2.49, p = 0.097, η_p_^2^ = 0.12) or choice latency (delay x impulsivity: F_(2,36)_ = 1.30, p = 0.284, η_p_^2^ = 0.06) (see Table 1).

**Table 1:** Accuracy and choice latency for LR choices for low impulsive (LI) and high impulsive (HI) animals during the delay discounting task.

			1 s delay	10 s delay	20 s delay
Accuracy (% correct)	LI	Control	86.67 ± 2.40	78.06 ± 4.23	81.39 ± 3.57
		Stress	80.28 ± 2.28	77.78 ± 2.88	66.67 ± 9.19
	HI	Control	87.00 ± 4.50	88.33 ± 2.83	69.67 ± 6.65
		Stress	77.67 ± 2.54	72.00 ± 7.71	60.33 ± 10.58
Choice latency (s)	LI	Control	0.79 ± 0.38	1.14 ± 0.53	1.16 ± 0.35
		Stress	0.59 ± 0.12	1.09 ± 0.22	1.28 ± 0.40
	HI	Control	0.60 ± 0.19	0.73 ± 0.18	1.11 ± 0.19
		Stress	0.51 ± 0.05	0.99 ± 0.19	1.76 ± 0.56

Data are the average across sessions (S1-2w) ± SEM (LI, n = 6; HI, n = 5, per group).

### Effects of ISD on delay discounting behavior

**Figure 3:**
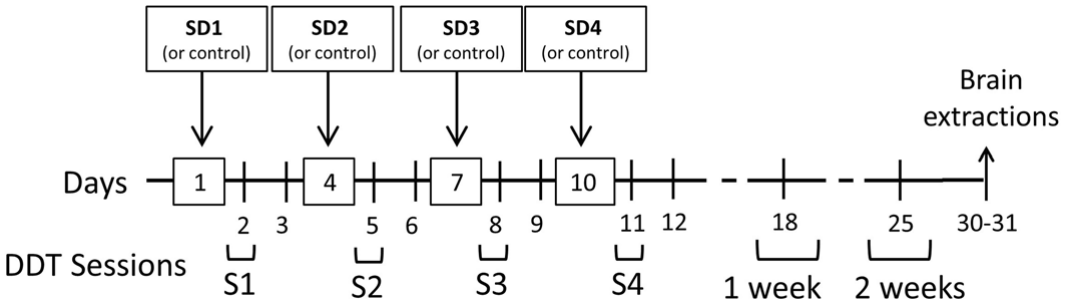
Timeline for the intermittent social defeat (ISD) stress protocol. Rats were exposed to social defeat (SD1-4) once every three days for ten days. Control animals were handled. The delay discounting task (DDT) sessions were performed 24 h after each social defeat episode (S1-S4) and 1 and 2 weeks after the last stress episode.

**Figure 4:**
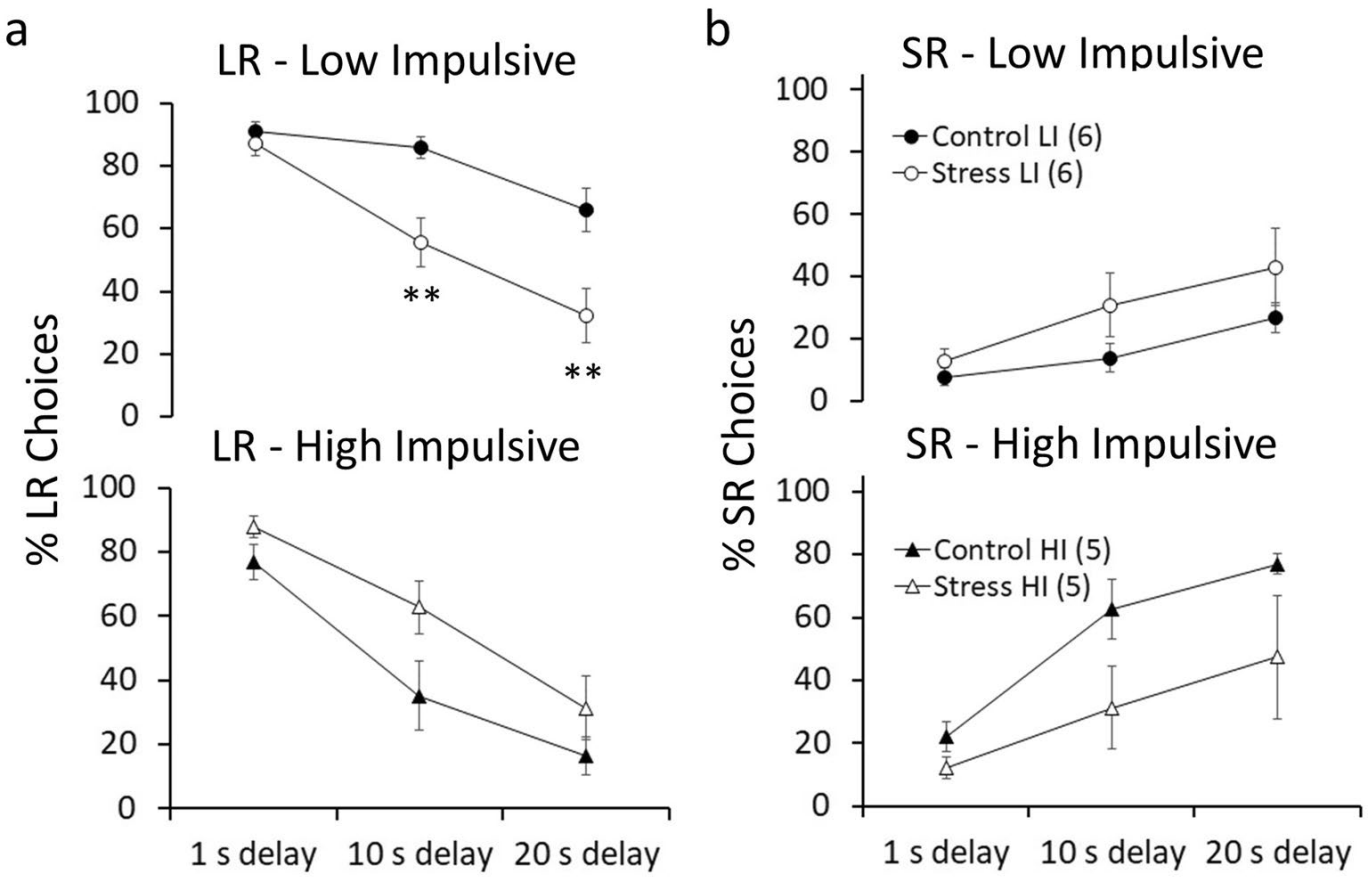
ISD stress decreases the preference for LR choices in low impulsive (LI), but not high impulsive (HI), animals. (**a**) LR and (**b**) SR choices at different delays for LR (1 s, 10 s and 20 s) comparing Control and Stress groups, for LI (top) and HI (bottom) animals. The delay for SR was always 1 s. Stressed LI animals showed decreases in LR choices with higher delays compared to Control (delay x group: F_(2,20)_ = 6.77, p = 0.006, η_p_^2^ = 0.40). This decrease was significant at both 10 s delay (F_(2,20)_ = 15.45, p = 0.003, η_p_^2^ = 0.60) and 20 s delay (F_(2,20)_ = 10.84, p = 0.008, η_p_^2^ = 0.52), but not 1 s delay (F_(2,20)_ = 0.74, p = 0.409, η_p_^2^ = 0.06). Stressed HI animals showed increases in LR choices compared to Control (group: F_(1,8)_ = 5.99, p = 0.040, η_p_^2^ = 0.42), although this effect was not delay-dependent (delay x group: F_(2,16)_ = 1.18, p = 0.330, η_p_^2^ = 0.12). Data represents the mean ± SEM. The mean values are the average of all the sessions (S1-2w) after starting the stress protocol (see Fig. 3). **p < 0.01 compared to Control after planned comparisons.

**Figure 5:**
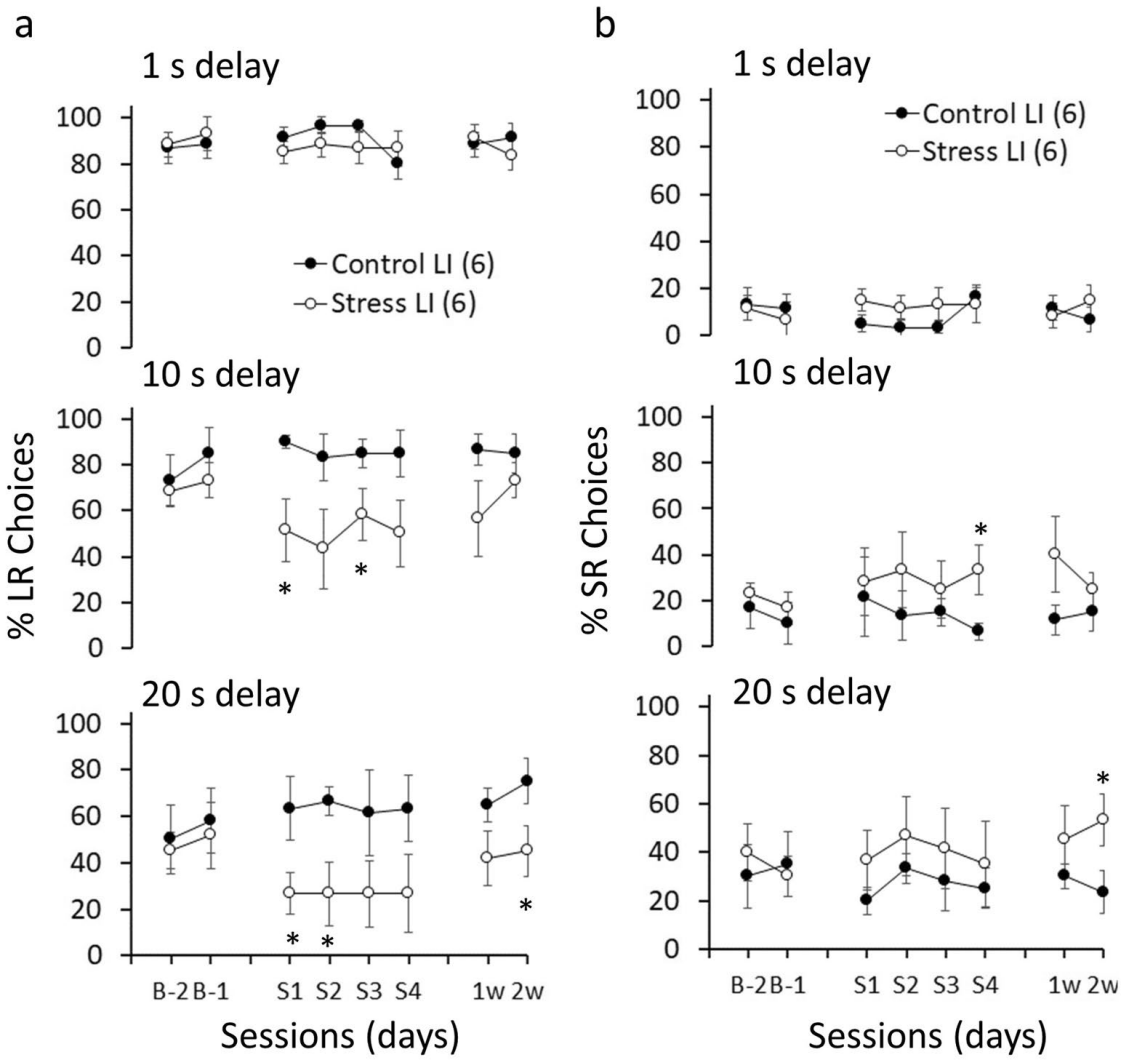
ISD decreases the preference for LR choices in low impulsive (LI) animals across sessions. (**a**) LR choices comparing control and stress groups at each delay for LR (1 s, 10 s and 20 s) and across sessions. Stressed animals showed significant decreases in LR choices at 10 s delay, S1 [F_(1,10)_ = 9.02, p = 0.013, η_p_^2^ = 0.47] and S3 [F_(1,10)_ = 5.04, p = 0.049, η_p_^2^ = 0.33], and 20 s delay, S1 [F_(1,10)_ = 6.05, p = 0.034, η_p_^2^ = 0.37], S2 [F_(1,10)_ = 8.47, p = 0.016, η_p_^2^ = 0.45] and 2w [F_(1,10)_ = 5.09, p = 0.048, η_p_^2^ = 0.33]. (**b**) SR choices comparing Control and Stress groups at each delay for LR and across sessions. Stressed animals showed significant increases in SR choices at 10 s delay, S4 [F_(1,10)_ = 6.53, p = 0.029, η_p_^2^ = 0.39], and 20 s delay, 2w [F_(1,10)_ = 5.54, p = 0.040, η_p_^2^ = 0.35)]. Data represents the mean ± SEM. *p < 0.05 compared to Control after planned comparisons.

The rats were tested in the DDT 24 h after each stress episode (S1-4) and 1 week (1w) and 2 weeks (2w) after the last stress episode (see Fig. 3). To analyze whether ISD changes delay discounting behavior, two- and three-way ANOVAs with repeated measures with group (Control, Stress) and basal impulsivity (LI, HI) as between subjects and delay (1 s, 10 s and 20 s) and/or sessions (S1-2w) as within subjects were performed to compare LR choices, SR choices, accuracy, choice latency and omissions. Figure 4 and Table 1 show the average across sessions (from S1 to 2w) of these variables (except omissions) and Fig. 5 and Table 2 show LR and SR choices per session for LI and HI animals, respectively.

**Table 2:** LR and SR choices for control and stress high impulsive animals (HI) at each delay for LR (1 s, 10 s and 20 s) and across sessions during the delay discounting task (see Fig. 3).

		B-2	B-1	S1	S2	S3	S4	1w	2w
LR choices (%)									
1 s	Control	90.0 ± 6.1	80.0 ± 11.7	84.0 ± 11.5	66.0 ± 8.3	82.0 ± 12.4	82.0 ± 4.1	68.0 ± 11.4	78.0 ± 6.5
	Stress	78.0 ± 5.4	94.0 ± 4.4	82.0 ± 7.4	90.0 ± 3.5*	84.0 ± 8.3	76.0 ± 8.3	94.0 ± 2.7*	100*
10 s	Control	62.0 ± 8.9	36.0 ± 10.3	44.0 ± 18.2	42.0 ± 19.8	40.0 ± 10.6	24.0 ± 7.5	28.0 ± 12.9	32.0 ± 19.1
	Stress	26.0 ± 9.0*	56.0 ± 5.7	70.0 ± 12.2	46.0 ± 7.5	66.0 ± 15.6	46.0 ± 11.5	74.0 ± 10.3*	74.0 ± 13.5
20 s	Control	20.0 ± 8.6	2.0 ± 2.2	12.0 ± 10.8	36.0 ± 15.2	12.0 ± 10.8	4.0 ± 4.4	14.0 ± 5.7	20.0 ± 12.2
	Stress	18.0 ± 8.9	20.0 ± 10.0	36.0 ± 15.2	20.0 ± 11.7	30.0 ± 10.6	44.0 ± 10.3*	34.0 ± 17.5	24.0 ± 9.0
SR choices (%)									
1 s	Control	10.0 ± 6.1	18.0 ± 10.2	16.0 ± 11.5	32.0 ± 8.9	14.0 ± 8.3	16.0 ± 2.7	32.0 ± 11.4	22.0 ± 6.5
	Stress	18.0 ± 5.4	6.0 ± 4.4	18.0 ± 7.4	10.0 ± 3.5*	16.0 ± 8.3	24.0 ± 8.3	6.0 ± 2.7*	0*
10 s	Control	34.0 ± 9.0	48.0 ± 14.3	62.0 ± 15.5	56.0 ± 21.1	42.0 ± 10.2	76.0 ± 7.5	72.0 ± 12.9	68.0 ± 19.1
	Stress	42.0 ± 17.4	26.0 ± 10.3	34.0 ± 22.2	34.0 ± 13.0	32.0 ± 16.3	40.0 ± 16.2	24.0 ± 11.5*	24.0 ± 14.4
20 s	Control	60.0 ± 11.7	78.0 ± 15.1	78.0 ± 12.9	62.0 ± 14.7	60.0 ± 16.9	96.0 ± 4.4	86.0 ± 5.7	80.0 ± 12.2
	Stress	38.0 ± 21.3	52.0 ± 24.3	46.0 ± 12.2	42.0 ± 21.3	50.0 ± 23.1	36.0 ± 18.2*	54.0 ± 21.6	56.0 ± 19.5

B-2 and B-1: basal; S1-S4: 24 h after each stress episode; 1w and 2w: 1 and 2 weeks after the last stress episode. Stressed HI animals showed significant increases in LR choices at 1 s delay, S2 [F_(1,8)_ = 8.72, p = 0.018, η_p_^2^ = 0.52], 1w [F_(1,8)_ = 6.14, p = 0.038, η_p_^2^ = 0.43] and 2w [F_(1,8)_ = 14.23, p = 0.005, η_p_^2^ = 0.64]; 10 s delay, B-2 [F_(1,8)_ = 9.96, p = 0.013, η_p_^2^ = 0.55] and 1w [F_(1,8)_ = 9.61, p = 0.015, η_p_^2^ = 0.54]; and 20 s delay, S4 [F_(1,8)_ = 15.68, p = 0.004, η_p_^2^ = 0.66]. Stressed HI animals showed significant decreases in SR choices at 1 s delay, S2 [F_(1,8)_ = 6.54, p = 0.034, η_p_^2^ = 0.45], 1w [F_(1,8)_ = 6.14, p = 0.038, η_p_^2^ = 0.43] and 2w [F_(1,8)_ = 14.23, p = 0.005, η_p_^2^ = 0.64]; 10 s delay, 1w [F_(1,8)_ = 9.60, p = 0.015, η_p_^2^ = 0.54]; and 20 s delay, S4 [F_(1,8)_ = 12.76, p = 0.007, η_p_^2^ = 0.60]. Data are the mean ± SEM (n = 5, per group). *p < 0.05 compared to Control after planned comparisons.

ISD decreased the preference for LR choices as the delay to reward delivery increases in LI rats, but not HI rats. As shown in Fig. 4a, stressed LI rats made fewer LR lever presses with higher delays compared to control LI rats (delay x group: F_(2,20)_ = 6.77, p = 0.006, η_p_^2^ = 0.40). Planned comparisons showed that this decrease was significant at both 10 s and 20 s delay, but not 1 s delay (see Fig. 4). In contrast, stressed HI rats made more LR choices compared to control HI rats (group: F_(1,8)_ = 5.99, p = 0.040, η_p_^2^ = 0.42), although this effect was not delay-dependent (delay x group: F_(2,16)_ = 1.18, p = 0.330, η_p_^2^ = 0.12). Figure 4b also shows that ISD increased and decreased SR choices in LI and HI rats, respectively (impulsivity × group: F_(1,18)_ = 7.79, p = 0.012, η_p_^2^ = 0.30), although this effect did not reach statistical significance when considered independently in LI (delay × group: F_(2,20)_ = 0.94, p = 0.406, η_p_^2^ = 0.08) or HI (delay x group: F_(2,16)_ = 1.50, p = 0.256, η_p_^2^ = 0.16) rats.

Table 1 shows that there were not significant differences between stressed and control rats, both LI and HI, in accuracy (LI, delay × group: F_(2,20)_ = 2.36, p = 0.120, η_p_^2^ = 0.19; HI, delay × group: F_(2,16)_ = 0.47, p = 0.632, η_p_^2^ = 0.05) or choice latency (LI, delay x group: F_(2,20)_ = 0.27, p = 0.759, η_p_^2^ = 0.02; HI, delay x group: F_(2,16)_ = 1.54, p = 0.245, η_p_^2^ = 0.16), with increasing delays. The number of omissions (i.e. not responding to any lever) during Free Choice trials was not significantly changed by group (F_(1,18)_ = 2.36, p = 0.142, η_p_^2^ = 0.11) or basal impulsivity (F_(1,18)_ = 0.09, p = 0.766, η_p_^2^ = 0.00) (data not shown).

Figure 5a shows that, in LI rats, there was a decreased preference for LR choices with higher delays in stressed rats compared to controls (delay × group: F_(2,20)_ = 5.74, p = 0.011, η_p_^2^ = 0.36), that started 24 h after the first stress episode and was maintained with no significant changes across sessions (delay x group x sessions: F_(14,140)_ = 0.51, p = 0.924, η_p_^2^ = 0.04). Planned comparisons showed group differences in some of the sessions at 10 s and 20 s delay (see Fig. 5). No significant differences were observed at 1 s delay between stressed and control rats. Planned comparisons also showed that stressed LI rats increased their preference for SR choices compared to controls in some of the sessions at 10 s and 20 s delay, but not 1 s delay (see Fig. 5b).

Table 2 shows the preference for LR and SR choices in stressed and control HI animals across sessions. Planned comparisons showed group differences in the preference for LR and SR choices in some of the sessions at 1 s, 10 s and 20 s delay (see Table 2). These group differences in both LR and SR choices across sessions were not delay dependent (LR: delay × group: F_(2,16)_ = 0.50, p = 0.613, η_p_^2^ = 0.05; SR: delay x group: F_(2,16)_ = 1.33, p = 0.292, η_p_^2^ = 0.14),

### Effects of ISD on PNNs and PVs in the mPFC

**Figure 6:**
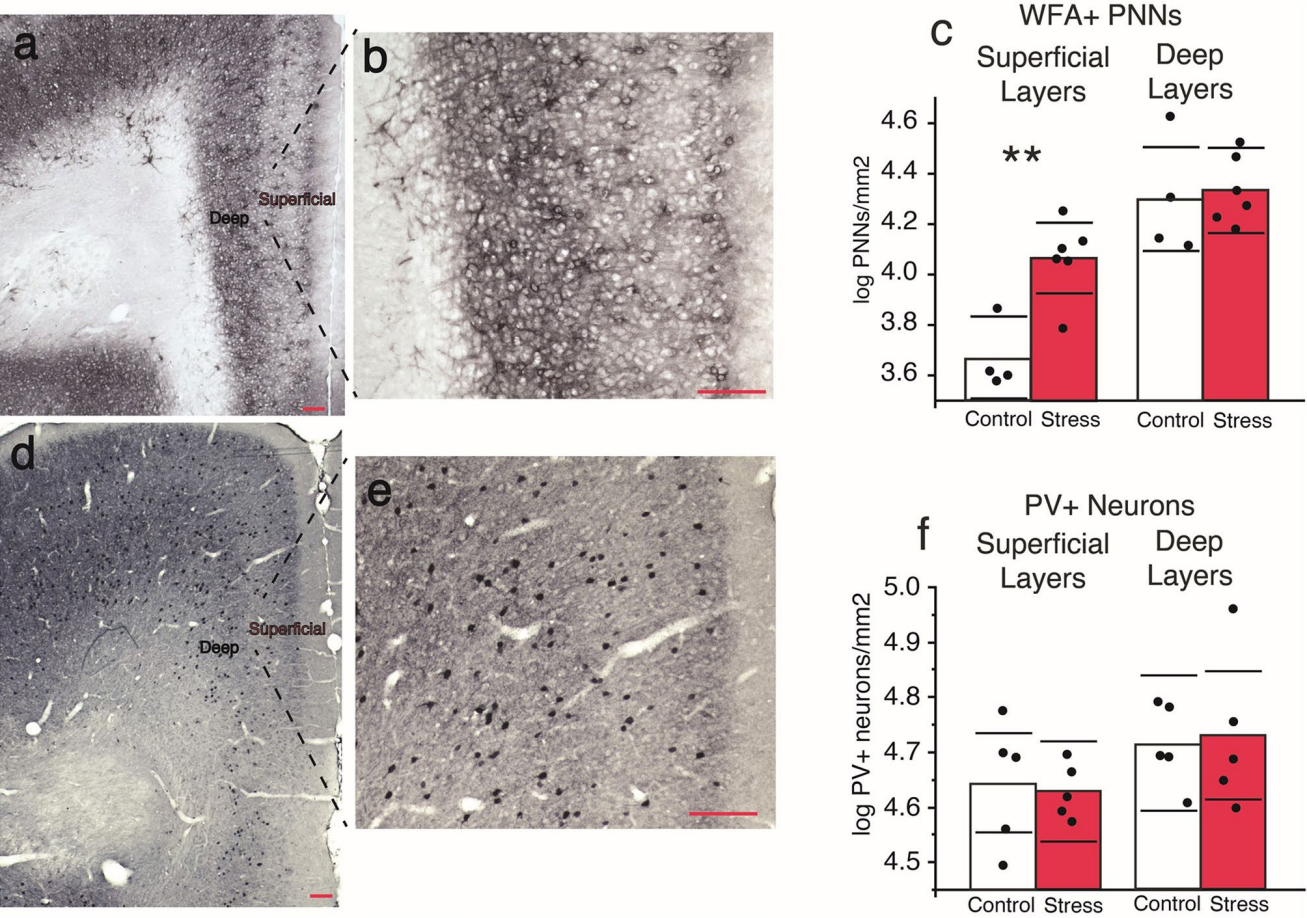
ISD increases the density of perineuronal nets (PNNs), but not parvalbumin GABA interneurons (PVs), in the superficial layers of the mPFC. (**a**,**b**,**d**,**e**) Microphotographs that highlight immunolabeling of PNNs and PVs in the cortical layers 2/3 of the prelimbic area. Density of PNNs (**c**) and PV cells (**f**) in superficial and deep cortical layers comparing Control (n = 4–5) and Stress groups (n = 5–6). Bars represent the mean and each dot a single animal. Horizontal lines indicate the 95% confidence interval of the samples. **p < 0.01 compared to Control after independent *t* test.

The density of PNNs and PVs immunolabeling was evaluated in the mPFC (prelimbic area) of stressed and control animals after behavior experiments. As shown in Fig. 6, ISD increased numerical densities of PNNs in the mPFC. Figure 6 also shows microphotographs indicating superficial and deep mPFC layers (Fig. 6a and d) and PNNs (Fig. 6b) and PVs (Fig. 6e) labeling. Figure 6c shows that PNN densities (PNNs/mm^2^) were significantly increased in the superficial (t(8) = 4.20, p = 0.003), but not deep (t(8) = 0.32, p = 0.753), cortical layers of stressed compared to control rats. In contrast, Fig. 6f shows that PV densities (PVs/mm^2^) were not different in the superficial (t(8) = 0.29, p = 0.778) or deep (t(8) = 0.25, p = 0.805) cortical layers of stressed compared to control rats.

We performed a linear regression analysis on the ISD group to assess whether increases in the density of PNNs was correlated to changes in LR choices at 10 s and 20 s delays. Changes in LR choices per animal were computed as the difference between basal LR choices (i.e. average of the last two training sessions) and LR choices after stress (i.e. average of all stress sessions from S1 to 2w). Pearson’s correlation coefficient did not show significant correlations between PNN density and LR choices after stress at 10 s (r = 0.25, p > 0.10) or 20 s (r = 0.32, p > 0.10) delay. Likewise, no significant correlations were found between these two variables before stress (i.e. basal) at 10 s (r = 0.34, p > 0.10) or 20 s (r = 0.30, p > 0.10) delay.

## Discussion

The present study shows that ISD increases delay discounting behavior and the density of PNNs in the mPFC. The effects of ISD on delay discounting depended on basal levels of impulsivity; ISD increased delay discounting in low impulsive, but not high impulsive, animals. Moreover, the effects of ISD were observed 24 h after the first stress episode and two weeks after the last stress episode. In this study we also show that ISD increases the density of PNNs in the superficial layers of the mPFC. These results suggest that a history of social stress enhances both choice impulsivity and cortical inhibition, and that these behavioral and neurobiological adaptations facilitate the transition from stress to SUDs as well as other psychiatric disorders such as major depressive disorder and anxiety disorders.

As shown, all rats decreased their preference for LR choices and increased their preference for SR choices with higher LR delays. These results agree with previous findings using similar DDTs in rats^32,47^ and humans^48^, and demonstrate that individuals devalue rewards that come with a delay. The decrease in the preference for LR choices (i.e. increased discounting rate) is used as an index of choice impulsivity and it is still debated how it relates to different aspects of value-based decision making such as reward or time processing^7,8^. By evaluating the number of correct responses during Forced Choice trials, we also show that accuracy was decreased at the highest delay. This finding agrees with previous studies^32^ and suggests that animals made more errors choosing the active lever when the time to reward delivery increases. We also found an increase in the latency to LR choices with higher delays indicating that a deliberative process is engaged when animals have to decide if they will wait more time for preferred rewards.

ISD disrupts delay-discounting behavior, and its effects depend on the basal levels of impulsivity. As previously reported by others^32,33,46^, we found that rats can be grouped in low impulsive and high impulsive according to their basal preference for LR choices. In low impulsive animals, ISD decreased the number of LR lever presses with higher delays (10 and 20 s) compared to controls, which suggests that ISD increases choice impulsivity. Importantly, both stressed and control animals preferred LR choices at the shortest delay (1 s) demonstrating that the effects of ISD depend on delay and are not related to disturbances in reward magnitude discrimination or motivation for food. Moreover, ISD did not change accuracy or choice latency, which indicates that ISD does not produce general alterations in cognitive (i.e. attention, working memory) or motor (i.e. motor impulsivity) behavior. The effects of ISD increasing delay discounting could be related to alterations in time processing (i.e. increase delay intolerance) or due to alterations in the subjective value of rewards, giving more value to immediate rewards in a context of higher uncertainty^47,49–51^.

Importantly, we assessed the effects of ISD on delay discounting behavior longitudinally, 24 h after each stress episode and up to two weeks after the last stress episode. This experimental design searches for potential time-dependent effects of stress on decision-making. In fact, by using a similar experimental design, we have recently shown that ISD produces time-dependent effects on effort-based reward-seeking behavior^23,45^. As shown, in low impulsive animals, ISD decreased the preference for LR choices 24 h after the first stress episode, which implicates that one social stress episode is enough to increase discounting behavior in the short term. Interestingly, adding more stressful events (up to four) did not further increase discounting rates but rather maintained this decision-making bias across sessions, up to two weeks after the last stress episode at 20 s delay. These results fit well the fact that ISD increases reward-seeking behavior (drug and natural rewards) in the long term^22,23^. Also, these results suggest that choice impulsivity after ISD predisposes animals to seek out rewards, which in the long term can facilitate the escalation of drug consumption.

In contrast to low impulsive animals, ISD increased the preference for LR choices in high impulsive animals. However, these effects did not depend on delay. In fact, when analyzed across sessions, high impulsive stressed animals showed more LR choices and fewer SR choices, compared to control animals, at all delays (1 s, 10 s and 20 s; see Table 2). Therefore, these effects might be related, at least in part, to aspects of reward processing different from intertemporal decision making. Previous studies have shown increases in the preference for LR choices after the administration of the pharmacological stressor, yohimbine, and suggested that stress can promote habitual responding during a decision-making task^52^. Psychostimulants and dopamine agonists have been also reported to decrease delay discounting behavior^25,53,54^. These studies suggest that, in high impulsive rats, ISD might increase reward magnitude sensitivity or tolerance for delayed rewards^25,50^ through changes in dopamine function. Elucidating the mechanisms by which ISD increases the preference for LR choices in high impulsive animals awaits further investigations.

The fact that the effects of ISD on delay discounting behavior depend on inherent impulsivity is remarkable and requires further discussion. Previous studies have shown that the inactivation of the orbitofrontal cortex^29^ and the nucleus accumbens^4^ produces different effects on delay discounting depending on the basal levels of impulsivity, and suggested that low and high impulsivity have different neurobiological substrates^55^. In support of this idea, evidence shows that a higher number of D2 dopamine receptors in the mPFC^46^ and the nucleus accumbens^49^ is associated with low and high impulsivity, respectively. Furthermore, a very recent study shows that basal impulsivity is associated with the activity of mPFC-nucleus accumbens projections^33^. Overall, these studies point to corticolimbic dopamine, and in particular, mPFC-nucleus accumbens projections, as main candidates in setting inherent impulsivity^47,53^. In this context, our data suggest that ISD induces adaptations in the activity of mPFC neuronal circuits to disrupt intertemporal decision-making.

We also show here that stressed animals had an increased density of PNNs in the mPFC compared to controls. PNNs are extracellular matrix structures that play a key role in neuronal plasticity and enhance the activity of PV cells, and, in turn, cortical inhibitory activity^41–44,56^. Previous studies have shown that chronic stress increases the expression of PNNs and the activity of PV interneurons in the mPFC, and that these effects are associated with stress-induced behavioral deficits^34,37^. Also, pharmacological and/or pharmacogenetic decrease in the expression of PNNs or PVs activity in the mPFC reverses the behavioral deficits produced by stress^38–40^. In line with these studies, the higher density of PNNs shown here suggests that an increased cortical inhibitory activity in the mPFC contributes to enhance choice impulsivity after ISD in low impulsive animals. A very recent study demonstrates that the inhibition of mPFC-nucleus accumbens projections increases choice impulsivity in a delay discounting task^33^. Based on this evidence, it is possible that ISD increases choice impulsivity by enhancing the inhibition over mPFC-nucleus accumbens projections. In support of this possibility, it has been recently shown that stress remodels cortical inhibitory circuits to regulate the activity of specific neuronal populations in the mPFC^36,57^.

We found that PNNs were increased only in cortical superficial layers (L2/3) but not in deep layers. Neurons in different layers receive different inputs and project to distinct targets, and their interconnections play crucial roles in intracortical processing^58^. Cortical layers 2/3 have a dense local connectivity of PV cells^59^ that can modulate working memory, behavioral flexibility, or social interaction in rats^60^. Previous studies have shown that chronic stress alters the activity of superficial cortical layers, which leads to ─or modulate─ stress-induced behavioral deficits^57,61^. This evidence fits well with our data and suggests that ISD alters delay discounting behavior by remodeling inhibitory circuits in superficial layers of the mPFC.

We did not find changes in the density of PV cells in the mPFC after ISD. Previous studies have shown that chronic stress increases, decreases or not change PV number in the mPFC^37,62^, and suggested that the effects of stress on PV depend on the type and intensity of stressors as well as the time when animals are stressed (i.e. early in life *versus* adulthood). But most importantly, PV density is not by itself an optimal index of PV activity, and in fact, some studies report changes in c-f*os* expression as a better marker of PV neuronal activation after stress^63^. Therefore, the fact that we did not observe changes in PV density after ISD does not necessarily indicate that ISD does not change PV activity and further studies are granted to elucidate this matter.

This study has limitations. First, we only utilized male rats. Previous studies have shown that the effects of ISD increasing drug self-administration are stronger in female compared to male rats^64^ which suggests that females are at higher risk to develop substance abuse after social stress. Sex differences have been also reported in decision-making behavior^65^. Based on these studies and our current work, we hypothesize that females will show higher choice impulsivity and PNNs densities in the mPFC compared to males after ISD. Second, previous work has shown that increases in delay discounting behavior depend on the order in which delays are presented and/or reward-related cues^29,54,66,67^. Regarding delay presentation, some studies have shown that the administration of amphetamine increases or decreases discounting behavior depending on the order of delay presentation^54,66^, and suggest that the effects of amphetamine reflect perseveration and not intertemporal decision-making deficits. Our results show that ISD increases discounting with increasing delays, which is the opposite of what was expected to be found if stressed animals persevere with a given response. Nonetheless whether the effects of ISD are modulated by the order of delay presentation or reward-related cues cannot be ruled out in this study. Third, we focused on the mPFC which is only one key area of a complex brain network^2,4,7^ that regulates the response to stress and intertemporal decision-making. Thus, it remains to be determined whether ISD also increases PNNs in other brain areas of this network such as the orbitofrontal cortex, the nucleus accumbens or the thalamus. Furthermore, our microscopy analysis was limited to two markers for inhibitory neurons. Future studies encompassing markers for additional GABAergic inhibitory neuron populations and PNN components will provide more insight into the neurocircuitry alterations in the mPFC of ISD animals. Finally, due to the small sample of brains analyzed, our results cannot determine whether increases in PNNs after ISD are different in low and high impulsive animals, or predict changes in LR choices, and therefore, further studies are granted to ascertain the relationship between stress-induced PNNs and choice impulsivity.

In summary, this study shows that ISD increases choice impulsivity in low impulsive animals and the number of PNNs in the mPFC. Given that ISD has been consistently shown to increase drug-seeking behavior weeks after stress, our results provide new insights into the behavioral and neurobiological mechanisms that govern the transition from stress to drug abuse. Our results also suggest that a better understanding of how a history of social stress disrupts intertemporal decision-making and remodels prefrontal inhibitory circuits will help predict, prevent, and treat SUDs. Recent studies have shown that PNNs and PVs in the mPFC regulate drug seeking and abuse^56,68,69^, and are associated with the beneficial effects of antidepressants^70^. In light of these studies, our data suggest that therapeutic strategies that target PNNs and PVs could reverse stress-induced choice impulsivity and ultimately prevent the transition to SUDs as well as other psychiatric disorders such as major depressive disorder and anxiety disorders^56,71–73^.

## Methods

### Animals

Twenty-four adult male Long Evans rats (Envigo, Indianapolis, IN) (375–425 g) were pair-housed in standard polycarbonate cages (45 × 24 × 20 cm) on a 12 h light/dark cycle (lights on at 9:00 P.M.). The sample size was based on our previous work^23,45^. In addition, four males (retired breeders; 500–600 g) and four females (300–325 g) (Long Evans) were utilized as residents during the stress protocol (see below). All experiments were performed during the dark phase when the animals are most active. The rats (except the residents) were placed on a mild food-restricted diet (15 g of chow per rat and day) two days before starting behavioral experiments^23^. All procedures were approved by the University of Mississippi Institutional Animal Review Board and were conducted in accordance with the National Institute of Health Guide for the Care and Use of Laboratory Animals.

### Experimental design

Two weeks after their arrival at the animal facility, all rats were handled, for at least three days, and then habituated to the operant chambers and trained in the DDT. Once animals reached stable performance, they were divided into two groups: Control (n = 11) and Stress (n = 11). Two animals that did not perform the task were discarded. After training in the DDT and one week before starting the stress protocol, animals were single-housed and remained single-housed for the duration of the study. The stress group was exposed to ISD following the protocol used in our previous studies^23,45^ and depicted in Fig. 3. The control group was taken to a new room the same amount of time and handled for 5 min. Rats were tested in the DDT 24 h after each stress episode and one and two weeks after the end of the stress protocol. Finally, the animals were sacrificed, and their brains extracted for immunohistochemistry assays.

### Delay-discounting task (DDT)

We utilized a DDT modified from Sackett et al.^32^ (Fig. 1A). The apparatus consisted of a sound attenuated operant chamber with two retractable levers (right and left) and a food trough in between them in the same wall (Coulbourn instruments). The chamber contained a house light that was on during the entire session. First, animals were trained to establish the lever–response contingency through a reward-shaping procedure using both levers (3–4 days) (dustless sugar pellets, 45 mg; Bio-Serv). Second, they were trained to discriminate between a large-reward (LR) lever and a small-reward (SR) lever (FR1, 30 min sessions). Pressing the LR lever delivered 3 sugar pellets while pressing the SR delivered 1 sugar pellet. This training phase lasted until they pressed the SR lever < 25% of the trials (5–6 days). Then, the DDT training started. The task consisted of 3 blocks of 20 trials each (60 trials total, < 60 min sessions). In each block, the first 10 trials were *Force Choice* trials in which the two levers were extended but the associated cue was lit in only one of them (one active lever). The right lever was associated with a SR (1 sugar pellet) and the left lever was associated with a LR (3 sugar pellets). This designation was counterbalanced among animals. The remaining 10 trials were *Free Choice* trials in which both levers were extended with the cue lights (two active levers), and animals were required to make a choice. Both levers were extended a maximum of 10 s and were retracted after animals made their choice. The three blocks were different in the delay to receive the large reward after pressing the LR: 1 s (block 1), 10 s (block 2) and 20 s (block 3). The pellet/s were delivered to the food trough, which light was turned on then. If animals did not press any lever within the 10 s both levers were retracted, and the trial was considered an omission. Animals were trained for 12 days until stable performance, which typically was accomplished after 5–7 days of training. A two-way ANOVA with repeated measures with group (pre-Control, pre-Stress) as between subjects, and delay (1 s, 10 s and 20 s) and sessions (6 last training sessions) as within subjects was performed to confirmed stable performance.

### Intermittent social defeat (ISD) stress

After stable performance in the DDT, rats were exposed to repeated ISD as in our previous work^23,45^ (Resident-Intruder paradigm, modified from Miczek et al.^22^). Rats (i.e. intruders) were housed individually one week before starting the stress protocol. Resident males (Long Evans retired breeders; 500–600 g) were housed in transparent PVC cages (H × L × W: 45 × 61 × 61 cm) with females for at least 10 days before starting the procedure. Female rats were previously sterilized by ligation of the oviducts (Envigo). Animals of the Stress group were exposed to social defeat once every three days for ten days (Fig. 3). Control animals were moved to a new room the same amount of time and handled for 5 min. Every social stress session started by removing the female rat from the resident cage at least 30 min before. Then, first, the intruder rat was placed in the cage with the resident male separated by a divider wall that contained wire mesh (allowing sensory exposure) ─both rats could see and smell each other─, for 10 min. Second, the divider wall was removed, allowing the rats to physically interact. The interaction was stopped when either 6 attacks were witnessed, the intruder was in supine position for 5 s, or 5 min had elapsed. The interaction time was extended up to 10 min if no attacks were witnessed during the first 5 min. To avoid injuries, the interaction was also stopped if an aggressive bite occurred. The latency to the first attack and the time in supine position were recorded. Third, the divider wall was reinserted, and the intruder remained in the cage for 10 more minutes. After this time, intruders returned to their home cages. Intruders were not exposed to the same resident more than twice.

### Immunohistochemistry

Five-six days after the last DDT session, the animals were deeply anesthetized [ketamine (100 mg/kg)/xylazine (15 mg/kg) mixture, i.p.] and then perfused with phosphate buffer (0.1 M) and 10% formalin. The brains were extracted, frozen with dry ice, and stored at -80 until slicing.

Serial free-floating tissue sections (spanning level 5 to level 10 of the Swanson Brain Maps 3.0 rat brain atlas (http://larrywswanson.com/?page_id=164) were carried through antigen retrieval in citric acid buffer (0.1 M citric acid, 0.2 M Na2HPO4) heated to 80 °C for 30 min and incubated in primary antibody monoclonal mouse anti-PV (1:10,000; P3088, lot #10K4846; clone PARV-19, ascites fluids; Sigma-Aldrich, St. Louis, Missouri) or biotinylated Wisteria floribunda agglutinin (WFA) lectin (1:1000, catalog #B-1355, Vector Labs), for 48 h at 4 °C. Immunoblot characterization for anti-PV P3088 showed a single band corresponding to 12 kD (information kindly provided by Sigma-Aldrich). WFA is an established marker for PNNs that binds to non-sulfated N-acetyl-D-galactosamine residues on the terminal ends of CSPGs.

Sections were subsequently in biotinylated secondary antibody horse anti-mouse IgG (1:500; Vector Labs, Inc. Burlingame, CA) for 2 h for PV followed by 2 h in streptavidin conjugated with horse-radish peroxidase for PV and WFA (1:5000 µl, Zymed, San Francisco, CA), and, finally, in nickel-enhanced diaminobenzidine/peroxidase reaction (0.02% diaminobenzidine, Sigma-Aldrich, 0.08% nickel-sulphate, 0.006% hydrogen peroxide in PB). All solutions were made in PBS with 0.2% Triton X (PBS-Tx) unless otherwise specified. Immunostained sections were mounted on gelatin-coated glass slides, coverslipped and coded for blinded quantitative analysis. All sections included in the study were processed simultaneously within the same session to avoid procedural differences.

Quantification was performed using a Zeiss Axioskop 2 Plus microscope interfaced with Stereo-Investigator v11. The superficial and deep layers of the prelimbic cortex were traced for an area measurement using a 10X objective, and PV neurons and WFA-labeled PNNs were quantified on 40X magnification using Stereo-Investigator v11.

### Data analysis

Two- and three-way ANOVA with repeated measures with group (Control, Stress) and basal impulsivity (LI and HI) as between subjects, and delay (Blocks 1, 2 and 3) and/or session (S1-2 weeks) as within subjects was performed to compare the following dependent variables: LR choice, SR choice, accuracy (i.e. number of correct responses in forced choice trials) and choice latency. Pair wise planned comparisons were performed to assess group effects using Bonferroni corrections. A median test including the average of LR presses across Blocks was performed to group animals into low impulsive and high impulsive. Numerical densities of immunoreactive cells were calculated as Nd = ∑N/∑V where N = sum of all cells counted in each region, and V is the volume of each region, calculated as V = ∑a ∙ z, where *z* is the thickness of each Section (30 µm) and *a* is area in µm^2^. Densities were log transformed prior to data statistical comparison between Control and Stress groups. A Student independent *t* test was used to evaluate the effects of ISD on PNNs and PVs in the mPFC. Pearson’s linear regression analysis was performed to assess correlation between PNN density values and LR choices. Statistical analysis was performed with SPSS software and the statistical significance was set at p = 0.05.

### ARRIVE

This study is reported in accordance with ARRIVE guidelines.

## Data Availability

The data and original contributions presented in this study are included in the manuscript and are available upon request to the corresponding author.
